# “Salicylic Acid Mutant Collection” as a Tool to Explore the Role of Salicylic Acid in Regulation of Plant Growth under a Changing Environment

**DOI:** 10.3390/ijms20246365

**Published:** 2019-12-17

**Authors:** Kamila Pluhařová, Hana Leontovyčová, Věra Stoudková, Romana Pospíchalová, Petr Maršík, Pavel Klouček, Anastasiia Starodubtseva, Oksana Iakovenko, Zuzana Krčková, Olga Valentová, Lenka Burketová, Martin Janda, Tetiana Kalachova

**Affiliations:** 1 Institute of Experimental Botany of the Czech Academy of Sciences, 16502 Prague, Czech Republicleontovycova@ueb.cas.cz (H.L.); stoudkovavera@seznam.cz (V.S.); pospichalova@ueb.cas.cz (R.P.); starodubtseva@ueb.cas.cz (A.S.); iakovenko@ueb.cas.cz (O.I.); krckova@ueb.cas.cz (Z.K.); burketova@ueb.cas.cz (L.B.); 2 Department of Biochemistry and Microbiology, University of Chemistry and Technology, 16628 Prague, Czech Republic; Olga.Valentova@vscht.cz; 3 Department of Biochemistry, Faculty of Science, Charles University in Prague, 11000 Prague, Czech Republic; 4 Department of Food Science, The Faculty of Agrobiology, Food and Natural Resources, The Czech University of Life Sciences Prague, 16500 Prague, Czech Republic; marsik@ueb.cas.cz (P.M.); kloucek@af.czu.cz (P.K.); 5 V.P. Kukhar Institute of Bioorganic Chemistry and Petrochemistry, National Academy of Sciences of Ukraine, 02094 Kyiv, Ukraine; 6 Genetics, Faculty of Biology, Ludwig-Maximilians-University of Munich (LMU), D-82152 Martinsried, Germany

**Keywords:** Salicylic acid, Arabidopsis mutants, light, growth, gene transcription

## Abstract

The phytohormone salicylic acid (SA) has a crucial role in plant physiology. Its role is best described in the context of plant response to pathogen attack. During infection, SA is rapidly accumulated throughout the green tissues and is important for both local and systemic defences. However, some genetic/metabolic variations can also result in SA overaccumulation in plants, even in basal conditions. To date, more than forty *Arabidopsis thaliana* mutants have been described as having enhanced endogenous SA levels or constitutively activated SA signalling pathways. In this study, we established a collection of mutants containing different SA levels due to diverse genetic modifications and distinct gene functions. We chose prototypic SA-overaccumulators (SA-OAs), such as *bon1-1*, but also “non-typical” ones such as *exo70b1-1*; the selection of OA is accompanied by their crosses with SA-deficient lines. Here, we extensively studied the plant development and SA level/signalling under various growth conditions in soil and in vitro, and showed a strong negative correlation between rosette size, SA content and *PR1*/*ICS1* transcript signature. SA-OAs (namely *cpr5*, *acd6*, *bon1-1*, *fah1/fah2* and *pi4kβ1β2*) had bigger rosettes under high light conditions, whereas WT plants did not. Our data provide new insights clarifying a link between SA and plant behaviour under environmental stresses. The presented SA mutant collection is thus a suitable tool to shed light on the mechanisms underlying trade-offs between growth and defence in plants.

## 1. Introduction

Salicylic acid (SA; 2-hydroxybenzoic acid) is a plant hormone (phytohormone) which plays a role in numerous plant physiological processes. It influences plant development, seed germination [1], cell cycle [2], flowering and responses to stresses [3]. In particular, the importance of SA has been reported in various abiotic stresses: response to high and low temperatures, humidity and drought, salt and osmotic stress [4] or responses to UV light [5]. However, since the 1990s, SA research has mainly focused on its role in immunity [6,7].

The role of SA in plant responses to stresses is generally studied using two approaches: a pharmacological approach using SA treatment on plants and a genetic approach that relies on mutants with modulated endogenous SA concentrations or SA-related signalling. These mutants can be either deficient in SA accumulation, or accumulating high levels of SA (in basal conditions and/or upon stimulation). The widely used SA-deficient lines are *sid2*, carrying a T-DNA insertion in *ISOCHORISMATE SYNTHASE 1* and thus showing lower production of SA upon pathogen attack [8]; or *NahG*, expressing bacterial SA hydroxylase that degrades SA to inactive catechol [9,10]. These mutants are generally more susceptible to pathogen attack, especially by biotrophs [11]. On the other hand, mutants synthesising higher basal levels of SA (SA-overaccumulating mutants; SA-OAs) tend to be more resistant to pathogens. The boom of using SA-OA mutants emerged with forward genetics approach, where EMS mutants were screened for spontaneous lesions and small sizes, which often coincided with high levels of SA and enhanced resistance to pathogens [12,13]. However, such resistance often correlates with general dwarfism [14]. This remains a critical disadvantage for potential use of SA pathway modification in agriculture, where the high yield is needed. Hence, the modulation of SA in terms of possible use in agriculture has to be carefully prepared. However, until now, the molecular mechanism of the trade-off is not fully understood yet.

The increased SA level in mutants could be triggered by distinct events (mutations). The SA-OA phenotype can be caused by gain-of-function mutation (typically activation of immune receptor) or by loss-of-function mutation (typically negative regulation of SA pathway). For example, *bon1-1* shows enhanced immunity and SA levels due to the mutation in the negative regulator of SNC1. This phenotype is thus reversed by introducing an *snc1-11* point mutation [15]. Particular interest has been devoted to mutants with altered phospholipid turnover/signalling and vesicular trafficking that were reported to have pleiotropic effects, often connected with SA accumulation. In particular, *pi4kβ1β2* mutation in phosphatidylinositol-4-kinases β1 and β2 was recently reported as an SA overaccumulator [16,17], or *exo70b1-1* [18]. *fah1/fah2* is deficient in fatty acid hydroxylase genes and also showed enhanced immune responses and a modulated sphingolipid profile [19]. Further characterisation of those lines will thus help in mechanistic understanding of the connections between phospholipid metabolism, vesicular trafficking and immunity in plants.

Here, we present a collection of *Arabidopsis thaliana* (hereinafter Arabidopsis) mutants with SA content altered in various ways: affected immunity-related signalling, modified vesicular trafficking and a directly altered SA biosynthesis/accumulation. As controls, we included crosses of SA-OAs with SA-deficient lines. We propose this collection as a tool to investigate the role of SA in plant growth regulation and stress tolerance.

## 2. Results

### 2.1. Cultivation Conditions Influence the Phenotype of the SA Collection Mutants

Our aim was to establish a collection from available sources of Arabidopsis mutants with alterations in the SA pathway with special attention to creating a group of highly diverse SA-OA mutants, and not only prototypic SA-OA ones. Thus, we selected two SA-deficient mutants (*NahG*, *sid2*), eight known or putative SA-OA mutants (*cpr5-1*, *acd6-1*, *pi4kβ1β2*, *fah1fah2*, *bon1-1*, *exo70B1-2*, *pmr4-1*, *edr2-6*), and four “reverted” mutants: SA-OA mutants crossed with the SA-deficient ones (*sid2pi4kβ1β2*, *NahGpi4kβ1β2*, *NahGedr2-6*, *bon1-1snc1-11*) (see Table 1).

**Figure 1:**
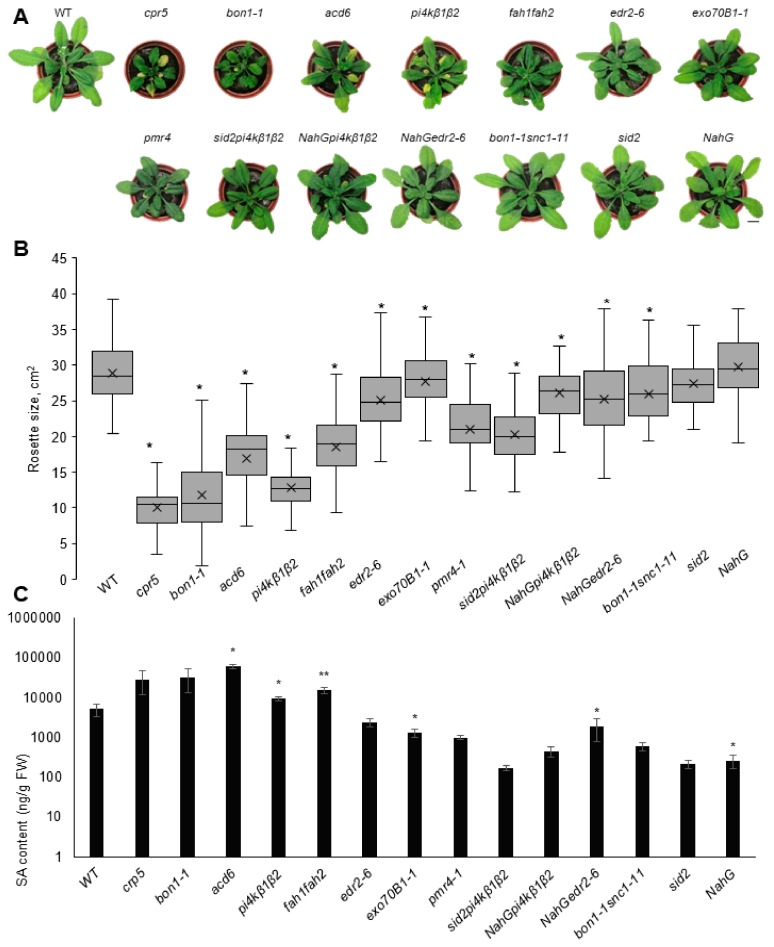
Rosette size and SA content of plants cultivated under long-day conditions. (**A**) Representative images of 4 week old plants cultivated at 22 °C, 16 h light/ 8 h dark. (**B**) Rosette size (area). Data are from three biological replicates, *n* ≥ 70. Central line of the boxplot represents the median occupancy, cross represents the mean, bottom and top edges of the box are 25 and 75% of distribution and the ends of whiskers are set at 1.5 times the interquartile range. (**C**) SA content in the leaves, *n* = 4. Data represent means + SEM, asterisks indicate variants different from WT, one-way ANOVA with Tukey’s HSD post hoc test, * *p* < 0.05, ** *p* < 0.01.

First we analysed the growth of selected mutants under long day (LD) and short day (SD) conditions. We initially focused on the 4 week old plants and analysed their rosette size and SA content (Figure 1; Supplementary Figure S1, Supplementary Table S2). Except for SA-deficient lines (*NahG* and *sid2*) and *exo70B1*, all others responded to LD condition with retarded growth. Due to their distinctive dwarf phenotypes (with an 85–50% reduction of rosette area compared to WT), we could clearly identify several SA-OA mutants: *cpr5*, *pi4kβ1β2*, *acd6* and *bon1-1* (Figure 1A,B). In SD conditions, the differences between mutants in growth were comparable to those under LD, although less important by absolute values (Figure S1A,B). In contrast, the differences in SA content were more pronounced at SD conditions. This could be due to a higher basal level of SA in the LD condition connected with a developmental stage; in LD conditions the plants started bolting at 3–3.5 weeks. In the “reverted mutants”, the SA level was decreased to the level of WT, which correlated with the WT-like rosette size (Figure 1 and Figure S1).

For better description of the effect of the growth conditions on dwarf phenotypes of SA-OAs, we focused on a subset of four mutants: WT, *sid2*, *pi4kβ1β2* and *sid2pi4kβ1β2*, previously used for studies of SA-related effects [17]. We compared the growth dynamics of this subset in several cultivation conditions often used in stress-related studies: SD, LD and greenhouse (Supplementary Figure S2). The *pi4kβ1β2* mutant appeared dwarfed under all conditions. Rosettes of *pi4kβ1β2* were smaller than in WT during the early developmental stages (2 week old seedlings), and the difference increased with time. Notably, the smaller rosettes did not result in a delay in development, since all plants started flowering simultaneously. Unexpectedly, *sid2pi4kβ1β2* grew bigger than *pi4kβ1β2* under all conditions but never reached the size of WT plants. This finding was surprising considering the previously published full reversal of growth in *sid2pi4kβ1β2* [25]. This finding indicates a high sensitivity of SA-related phenotypes to cultivation conditions.

Particular attention was directed to the *pmr4* mutant, deficient in callose synthase CALS12 and first described as POWDERY MILDEW RESISTANT 4 [24]. Under both SD and LD, the plants appeared slightly smaller than WT; however, no increase of SA was detected (Figure 1, Supplementary Figure S1, Supplementary Table S2). To the best of our knowledge, this is the first report on the (comparative) determination of the SA content in *pmr4* mutants, as the previous studies claimed a constitutively activated SA pathway based on SA-related gene transcription and resistance to pathogens [24].

### 2.2. SA-Related Gene Transcription Varies in Different Growth Conditions

**Figure 2:**
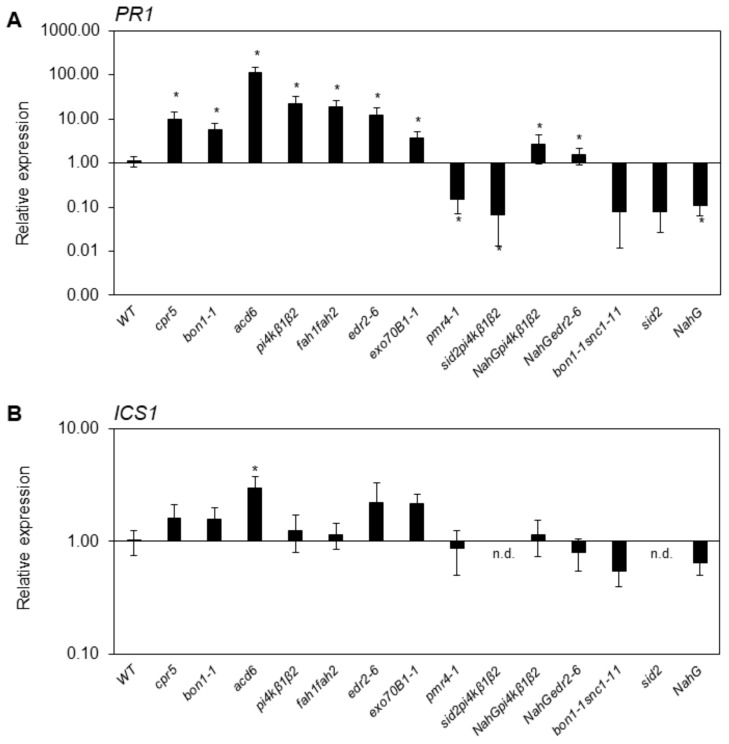
Transcription of *ICS1* and *PR1* in soil-grown plants cultivated under LD conditions. Samples were collected from four 4 week old plants. Values were normalized to WT at the respective conditions. *TIP41* was used as a reference gene. Data represent means + SEM, asterisks indicate values different from WT, *t*-test, * *p* < 0.05, *n* = 4.

We analysed the SA marker genes’ (*PR1* and *ICS1*) transcription in soil-grown plants under SD and LD conditions. In most cases, the expression of the *PR1* gene coincided with small rosettes and a higher level of SA (Figure 2, Supplementary Figure S3). Generally, our results confirmed those from the studies wherein the mutants were first described. Under SD conditions, the differences between mutants were more pronounced both in *PR1*/*ICS1* transcription and in SA content. As gene transcription data were normalized to WT in both conditions, and WT at LD had almost 5 times higher SA content than in SD, that might have strongly affected basal *PR1* level.

To link SA-related signatures to the physiological state of plants, we measured photosynthetic activity. As reliable parameters of photosynthetic state, we chose quantum yield of PSII photochemistry (QY) and non-photochemical quenching (NPQ) [27]. Increase of NPQ can occur as a result either of the processes that protect the leaf from damage or of the damage itself [28]. The changes in NPQ were observed in several studied mutants (Supplementary Figure S4). NPQ at steady state (NPQ_Lss) was decreased in *snc1-1* compared to WT, but increased in several other mutants from the collection. These mutants were mainly “immune-related” mutants *cpr5* and *bon1-1* and “trafficking” mutants *pi4kβ1β2* and *exo70B1-1*. Notably, the values were similar to those in “reverted” mutants *sid2pi4kβ1β2*, *NahGpi4kβ1β2* and *bon1-1snc1-11*, suggesting SA-independent origin of NPQ_Lss increase. The SA-deficient mutants also showed slight (by 10%) increase in NPQ_Lss, indicating SA independency. Maximum quantum efficiency of PSII photochemistry (QY_max) was stable among all studied mutants. Generally, the analysis of photosynthetic parameters did not reveal any drastic differences between the selected mutants in basal conditions.

### 2.3. Overaccumulation of SA Increases High Light Sensitivity in In-Vitro-Grown Seedlings

**Figure 3:**
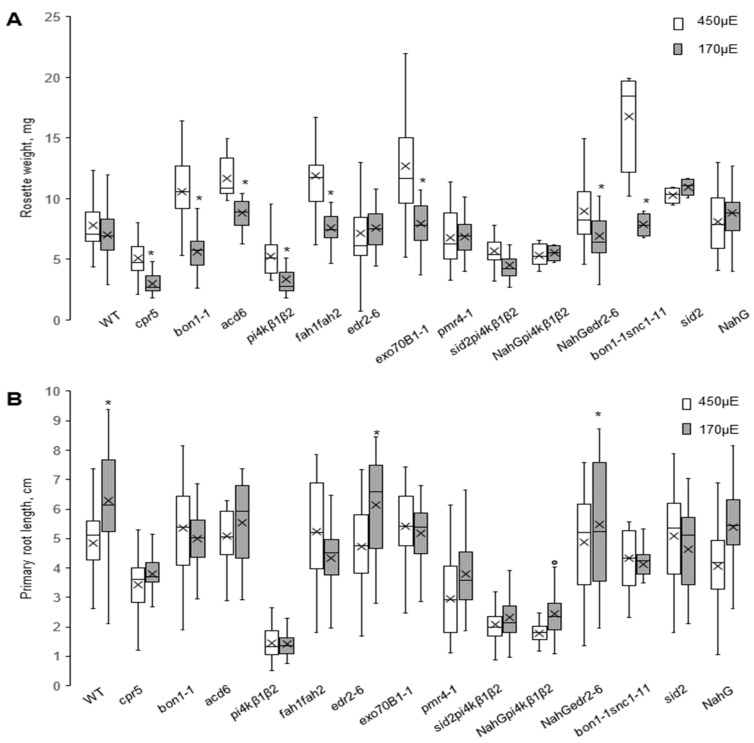
In vitro growth of SA collection mutants under different light intensities. Two week old seedlings were cultivated on ½ MS medium under 450 μE.m^−2^.s^−1^ or 170 μE.m^−2^.s^−1^ under 12 h light /12 h dark photoperiod. (**A**) Rosette weight. (**B**) Primary root length. Data represent four biological repetitions; at least 10 seedlings were measured for each variant in each biological repetition. Central line of the boxplot represents the median occupancy, cross represents the mean, bottom and top edges of the box are 25 and 75% of distribution and the ends of whiskers are set at 1.5 times the interquartile range, asterisks indicates variants different from those for the 450 μE.m^−2^.s^−1^ intensity the same genotype, * *p* < 0.01, *t*-test.

To investigate the behaviour of the SA mutant collection under in vitro conditions, we switched to the in vitro setup often used for the study of developmental defects. Continuous illumination of the roots, though widely used in research, can cause diverse effects on the phenotype, including spontaneous production of reactive oxygen species [29]. To study the reliability of our collection for root phenotyping, we studied seedling growth in vitro upon different light conditions. Seedlings were grown in vertically placed Petri dishes under LD light regime and at two light intensities, 450 μE.m^−2^.s^−1^ and 170 μE.m^−2^.s^−1^. Rosette weight and primary root length were evaluated at 11 dpg (Figure 3). While the rosette weight of WT plants did not change in response to light intensity, the mutants exhibiting high changes in SA content showed more intensive growth under 450 μE.m^−2^.s^−1^ light. Interestingly, such an effect was not evident within the majority of the reverted group, except for *bon1-1snc1-11*. SA-deficient genotypes and *pmr4-1* grew similarly at both light intensities, thus highlighting the role of SA in this effect (Figure 3A).

The growth of the primary roots was moderately inhibited by high light intensity in WT and also in both genotypes carrying the *NahG* transgene; however, the roots of other mutants were almost insensitive to different light conditions (Figure 3B). On the other hand, some mutants with pronounced dwarf rosette phenotypes had roots of the same size as WT plants (*bon1-1, acd6*). In contrast, in the *pi4kβ1β2* mutants, which had both small rosettes and short roots in all studied setups, the mechanisms regulating root and rosette growth inhibition seemed to be distinct. Indeed, while the rosette sizes were particularly restored by preventing SA accumulation (*sid2pi4kβ1β2*, *NahGpi4kβ1β2*), the roots remained small, indicating the SA-independent character of the phenotype (Figure 3B). To quantify this in time-course and to further investigate the effect of light regime on root growth, we focused on the phosphatidylinositol-4-kinase- related subset (WT, *sid2*, *pi4kβ1β2* and *sid2pi4kβ1β2*). First, we measured root elongation in kinetics at the light intensity corresponding to 170 μE.m^−2^.s^−1^ (Supplementary Figure S5A). The root defects caused by *pi4kβ1β2* mutations appeared at the very early germination stages and this effect was enhanced in time. To confirm the light independency of the phenotype, we also analysed root length in a semi-dark setup, modified from Silva-Navas et al., 2015 [30]. The Petri dishes were placed in dark plastic covers to shadow roots (Supplementary Figure S5B). In both setups, the growth kinetics were comparable: in the dark setup, *pi4kβ1β2* roots were about 3 times shorter than WT at 4 dpg, and about 5 times shorter at 8 dpg. Furthermore, *sid2pi4kβ1β2* roots were about 20% longer than *pi4kβ1β2* at 4 dpg and 50 % longer at 8 dpg. However, while the difference between *pi4kβ1β2* and WT remained stable in the light setup (up to 6 times at 8dpg), the difference between *pi4kβ1β2* and *sid2pi4kβ1β2* was more pronounced, up to 30% at 4 dpg and up to 200% at 8 dpg. This confirmed the SA-dependent sensitivity to light in in vitro growth conditions, and it also means that the light regime should be seriously considered in various types of experiments, especially those connected with SA.

With the SA collection, we were able to show that the regulation of the rosettes and root size is independent: the SA content mostly influenced the aboveground plant part, while the root length corresponded to SA-independent phenotype. Indeed, while *bon1-1* and *pi4kβ1β2* mutants were similar in terms of rosette growth, the roots of *bon1-1* were twice longer than that of *pi4kβ1β2* at both light intensities (Figure 1B and Figure 3A,B).

### 2.4. Salicylic Acid’s Effect on the Root Growth and Shoot Growth is Distinct

**Figure 4:**
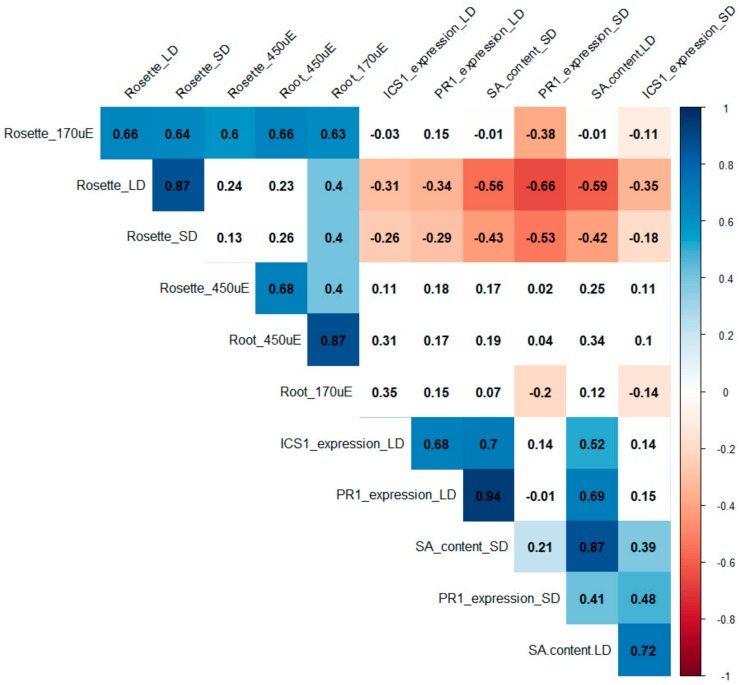
Correlation table of SA effects on growth. The matrix was built using the Pearson correlation for 12 parameters (rosette size, SA content and SA-related gene expression (*ICS1* and *PR1*) for soil-grown plants under short-day (SD) and long-day (LD) conditions; and rosette weight and primary root length for in vitro grown plants grown under an LD photoperiod at 450 uE or 170 uE light intensity). Measurements were taken for 15 genotypes (listed in Table 1). Data are from three biological repetitions for each variant. Positive correlations are displayed in blue and negative correlations in red. Correlation coefficients are indicated. Only results that passed the 0.05 threshold for significance are displayed in colour.

To evaluate the behaviour of the presented SA collection in various growth setups, we performed a correlation analysis among all studied parameters: rosette size, SA content and expression of *ICS1* and *PR1* genes in soil-grown plants under two light regimes; and rosette weight and primary root length of seedlings cultivated in vitro under two light intensities. Putting together data of three biological repeats of all 15 genotypes in the collection, we quantified Pearson correlations (Figure 4).

The correlation table provided several clear outcomes: the rosette size of plants grown in soil negatively correlated with SA content accompanied with *PR1*/*ICS1* upregulation, which has been abundantly shown in previous studies [31]. Rosette growth correlated positively in all conditions. In contrast, the root growth in in vitro conditions was SA-independent (Figure 4). Generally, the rosette growth correlated with root growth, despite the above-mentioned difference between *bon1-1* and *pi4kβ1β2.* Interestingly, only seedlings grown under the 170 μE.m^−2^.s^−1^ intensity strongly correlated with rosette growth of soil-grown plants, suggesting that particular attention needs to be paid to light intensity while comparing data obtained in different growth conditions.

## 3. Discussion

SA plays a role in many fundamental processes in plants. Nowadays, it is probably the best characterised phytohormone in connection with plant immunity. A great tool which have provided insight into SA signalling pathways and their roles, especially in Arabidopsis, is SA-OA mutants. Interestingly, some of the initially described immunity-related mutants later appeared to have altered SA metabolism/signalling [31]. Changes in SA levels have a very strong impact on plant growth, and the majority of known SA-OA mutants are dwarfs. Because of their clearly distinguishable growth phenotypes, SA-OA mutants have been successfully used to find new components of plant immunity in forward genetic screening [32], in evolutionary studies [33,34] and in studies of ubiquitination cascades [35]. Growth inhibition of SA-OA has been used as a marker of an activated immune state in heat stress experiments: SA-OA mutants exhibit dwarf phenotypes under 22 °C, but have WT-like phenotype under 28 °C [36]. Although they have been studied for more than 30 years, SA-OA mutants still display many features that lack mechanistic explanation. One of them is the impact of cultivation conditions on SA-regulated growth, which has never been extensively studied.

To gain a complex understanding of the connection between growth and SA, we created a collection of 14 SA-modulated Arabidopsis mutants in a Col-0 background. We collected mutants from already published studies, including prototypic SA-deficient mutants *sid2* and *NahG* and prototypic SA-OA mutants *bon1-1*, *cpr5-1* and *acd6-1*. Additionally, we included recently described SA-OAs connected with lipid signalling, *pi4kβ1β2* and *fah1fah2*, and mutants associated with SA signalling based on gene expression analysis and pathogen assays, *edr2-6*, *pmr4-1*, *exo70b1* (Table 1). To complete the picture, we included three SA-OA “reverted lines”, in which SA-OA mutants were prevented from accumulating a high SA level by affected biosynthesis (*sid2pi4kβ1β2*) or accumulation (*NahGpi4kβ1β2* and *NahGedr2-6*). All the selected mutants have been reported as having altered resistance to pathogens [31].

While analysing mutant phenotypes under various conditions, it is often difficult to distinguish between “typical” immune response and “just pleotropic” effects caused by mutation. A good example is the *pi4kβ1β2* mutant with impaired vesicle trafficking, which is a ubiquitous process that affects almost everything in plant cells [22]. We studied the SA-(in) dependent effects in *pi4kβ1β2*, showing that resistance to adapted pathogens is strictly SA-dependent, but callose production is SA-independent [17]. By creating this type of collection, we wanted to be able to compare more mutants with modulated SA patterns under exactly the same experimental conditions. For this purpose, we started with characterisation of the plant growth under short-day (SD) and long-day (LD) conditions. In general, our data confirmed previously published data that SA-OA mutants exhibit dwarf phenotypes (Figure 1A,B and Figure S1A,B). The SA content negatively correlated with rosette size (Figure 1 and Supplementary Figure S1,S4) which has been previously shown in literature [31]. However, the SA measurement also revealed that *edr2* and *exo70b1* are SA-OAs only under short-day conditions and *pmr4* is not SA-OA at all. This is particularly important as *edr2* and *pmr4* mutants have previously been described to accumulate high SA under biotic stress conditions, which thus suggests enhanced SA pathways at basal conditions as well. As expected, higher SA content in basal conditions was shown for *edr2* [23], but, surprisingly, we were unable to find in the literature any SA measurement for the *pmr4* mutant, although it is generally referred to as the one with constitutively induced SA pathways [24]. Again, SA marker genes are highly enhanced in *pmr4* under biotic stress conditions [37]. This statement is based on *PR1* gene expression, but not on SA level itself. In our setup, *PR1* expression was not highly induced even in basal conditions.

First, we characterised the SA collection‘s growth, *PR1/ICS1* transcription and SA content in plants cultivated in soil under LD and SD conditions (Figure 1 and Figure S1). Interestingly, in WT plants PR1 transcription was 5 times higher under LD than SD. That coincides with the fact that under LD conditions, plants tended to bolt at the age of 4 weeks. The induction of flowering is also associated with an increase in SA content and vice versa—SA treatment can trigger flowering [38]. No drastic changes in photosynthesis efficiency were detected (Supplementary Figure S3). For the full set of analysed mutants, we observed a negative correlation between rosette size and SA content under both LD and SD conditions. However, our data suggest that growth phenotype related to SA content would be better investigated under LD conditions. On the other hand, differences in SA content and gene transcription of SA marker genes were more pronounced under SD conditions. In comparison with the literature, our data showed that the mutants with modulated SA pathways were very sensitive to growth conditions. In terms of growth size, this could be clearly seen in the WT, *sid2*, *pi4kβ1β2* and *sid2pi4kβ1β2* subsets. This has been previously used to distinguish between SA-dependent and SA-independent effects of *pi4kβ1β2* deficiency [17,25]. Interestingly, in Šašek et al. (2014) [25], we showed that crossing of *pi4kβ1β2* with *sid2* led to a fully reverted phenotype when plants were grown in soil for 4 weeks. In our current cultivation conditions, we were not able to fully revert the growth (Figure 1A). We studied this in more detail under three distinct growth conditions. Two were in climate chambers with 8 h/16 h (light/dark) (short day) or 16 h/8 h (light/dark) (long day) and one was in greenhouse conditions. In all setups, *sid2pi4kβ1β2* was smaller than WT. In SD, *sid2pi4kβ1β2* had a size comparable even to *pi4kβ1β2* (Figure S2). The data of *ICS1* expression showed that the *ICS1* mutation was functional in both *sid2* and *sid2pi4kβ1β2* lines (Figure 2 and Figure S2). Such behaviour can affect data interpretation and highlights the importance of checking SA levels while studying pleiotropic phenotypes, especially in a newly discovered mutant lines.

Early studies of SA mutants were mostly done on the rosettes (leaves) of soil-grown plants, while in recent years, the usage of in-vitro-grown seedlings as a model system has been rapidly increasing. The induction of the SA pathway has been shown during infection with root pathogen *Trichoderma* [39]. The sensitivity of Arabidopsis roots to SA treatment was recently demonstrated by a proteomics and metabolomics approach using SA-altered mutants [40]. Furthermore, the role of SA in root morphogenesis was recently shown by Pasternak et al. 2019 [41]. These authors reported SA treatment to modulate root meristem patterning by affecting auxin signalling in a concentration-dependent manner. However, no mutants with modulated SA were used in the study and the usage of only a pharmacological approach often raises questions about appropriate controls. We believe that the SA collection could be a helpful tool to continue studies of hormonal cross-talk in Arabidopsis roots. Here, we showed that root growth in the SA mutant collection is highly variable (Figure 3), and is not correlated with SA levels or SA marker gene expression in the rosettes of soil-grown plants (Figure 4). A clear example is the comparison of *bon1-1* phenotype (small rosette and almost WT-size roots) to *pi4kβ1β2,* which also had small rosettes but impaired root growth (Figure 1 and Figure 3). Our data confirmed the critical role of PI4Kβ1β2 for root growth (Figure 3) [16,25]. To precisely analyse the SA role in seedlings’ sensitivity to light, we used the subset of WT, *sid2*, *pi4kβ1β2* and *sid2pi4kβ1β2*. We grew plants in a light growth setup (roots were exposed to light) and dark growth setup (roots were shadowed by placing in dark chambers). The SA-deficient mutant *sid2* grew similarly to WT under dark conditions, but slower in the light setup, and both *pi4kβ1β2* and *sid2pi4kβ1β2* roots grow slower, while the difference between them was more pronounced in a light setup (Figure S4).

As mentioned above, SA-OA mutants are indispensable in studies of SA-related immunity. Additionally, SA’s role in biotic stress was also shown via SA-OA involvement in response to abiotic stresses. In particular, the role of SA in cold stress was shown using *acd6* [21], *cpr1* and *pi4kβ1β2* [42]; in potassium stress by using *cpr5* [43], in response to drought and ABA treatment by using *cpr5* and *acd6* [44,45], and in sugar sensing by using *acd6* and *cpr1* [46]. Here, we tested the behaviour of these mutants under distinct growth conditions and under moderate abiotic stress in vitro (distinct light intensities). Surprisingly, the SA-OA mutants *cpr5*, *acd6*, *bon1-1*, *fah1/fah2* and *pi4kβ1β2* tended to form bigger rosettes under higher light intensities, while size of the WT rosettes was not affected (Figure 3). This indicates that SA makes plant more sensitive to high light conditions. In contrast, WT root growth was inhibited by high light but the roots of the above-mentioned SA-OA mutants were not affected (Figure 3B). These findings suggest an opposite effect of light on rosette and root growth.

The trade-off between immunity and growth has been widely discussed [14,47,48,49]. Our data present a robust quantitative background for this. We have shown strong negative correlation between SA levels and *PR1/ICS1* transcript signature with rosette size, but no correlation with root growth. This is important to take into account while planning phenotyping of mutants on different scales, and also confirms the suitability of putative SA-OA mutants for studies of root growth without impact of SA itself.

## 4. Materials and Methods

### 4.1. Plant Material and Growth Conditions

*Arabidopsis thaliana* Col-0 was used as a wild type (WT), and the collection consisted of following mutants (see Table 1): *crp5 (SALK_071947)*, *bon1-1 (SALK_123132)*, *acd6 (SALK_059132), pi4kβ1β2* (SALK_040479/SALK_09069), *fah1fah2* (SALK_094443, SALK_033090), *exo70B1-1 (CS410875)*, *edr2-6 (CS66944); NahG* [9], *sid2-3* (*SALK_042603*); *bon1-1snc1-11* (SALK_047058, SALK_123132)*, NahGpi4kβ1β2*, *sid2pi4kβ1β2* [25] and *NahGedr2-6 (CS66944*). Prior to experiments, all seeds were propagated for one generation under the same conditions and genotyped as described in the literature (see Table 1).

Plants were grown in two main setups: in a cultivation substrate (soil) (a), and in vitro (b). For both setups, seeds were sterilized in 1.6% sodium hypochloride (30% of SAVO^®^, Unilever) solution with 0.02%TWEEN20 (Sigma Aldrich, St. Louis, Missouri, USA). Stratification for 2 days at 4 °C in dark conditionswas applied to break dormancy. (a) In soil: seeds were transferred to pots with substrate tablets (Jiffy, Kristiansand, Norway and grown in cultivation chambers (Snijders, Drogenbos, Belgium at 22 °C day temperature, 65–70% humidity and 16 h light/ 8 h dark (LD) or 12 h light/ 12 h dark (SD). After one week, the seedlings were replanted to one plant per pot. Four week old plants were used for analysis. (b) In vitro: seeds were germinated for 3 days in Petri dishes containing a half-strength Mirashige–Skoog medium (½ Murashige–Skoog basal salts (Duchefa), pH = 5.7) supplemented with 1% sucrose and 0.8% plant agar (Duchefa, Haarlem, Netherlands. At 4 days, seedlings were aseptically transferred to new plates and cultivated in a vertical position in cultivation chambers (Snijders) at 22 °C under long-day light conditions. After one week (11 days after germination), the Petri dishes were scanned (Epson Perfection V700 Photo, Suwa, Japanc), the root length was measured and the rosettes were cut and weighted. Root length was measured by FiJi software [50].

To investigate the effect of light on root development, the seedlings were grown under continuous exposure to light at different intensities, 450 μE.m^−2^.s^−1^ or 170 μE.m^−2^.s^−1^, or in the dark (plates were put in black chambers to shadow roots, Supplementary Figure S5B). To investigate the kinetics of root growth, the primary root length was monitored daily from 4 dpg to 8 dpg and measured using FiJi [50]. At least 10 roots were analysed for each condition.

### 4.2. Plant Phenotyping

Rosette size of soil-grown plants and primary root length of seedlings were measured by FiJi (area tool) [50]. Rosette weight of 11 day old seedlings was determined using analytical scales.

### 4.3. SA Concentration Measurements

Leaf tissue was collected from three plants (approximately 100 mg, three 6 mm discs from three leaves) in Eppendorf tubes with 1 g ceramic beads and frozen in liquid nitrogen. Hormone extraction procedure and salicylic acid content measurement were done as in [51]. Briefly, frozen samples were homogenized in tubes with silica beads using a FastPrep-24 instrument (MP Biomedicals, CA, United States) with extraction reagent methanol/water/formic acid (15:4:1, *v*/*v*/*v*) supplemented with stable-isotope-labelled 13C-SA internal standards. Extracts were subjected to solid phase extraction using Oasis MCX cartridges (Waters Co., Milford, MA, United States) and eluted with methanol. The eluate was evaporated to dryness and dissolved in 15% acetonitrile/water (*v*/*v*) immediately before the analysis. Quantification was performed on an Ultimate 3000 high-performance liquid chromatograph (UHPLC, Dionex; Thermo Fisher Scientific, Waltham, MA, United States) coupled to a IMPACT II Q-TOF ultra-high resolution and high-mass-accuracy mass spectrometer (HRAM-MS; Bruker Daltonik, Bremen, Germany). Separation was carried out using an Acclaim RSLC 120 C18 column (2.2 m, 2.1 × 100 mm; Thermo Fisher Scientific, Waltham, MA, United States) mobile phase consisting of 0.1% formic acid (A) and methanol (B) by gradient elution. The full-scan data were recorded in negative electrospray ionization (ESI) mode.

### 4.4. Gene Transcription Analysis

Total RNA was extracted as in [51]. Briefly, plant tissue was frozen in liquid nitrogen. The tissue was homogenized in plastic Eppendorf tubes with silica beads using a FastPrep-24 instrument (MP Biomedicals, USA). Total RNA was isolated using Spectrum Plant Total RNA kit (Sigma-Aldrich, St Louis, Missouri, USA) and treated with a DNA-free kit (Ambion, Austin, Texas, USA). Subsequently, 1 μg of RNA was converted into cDNA with M-MLV RNase H– Point Mutant reverse transcriptase (Promega Corp., Madison, Wisconsin, USA) and an anchored oligo dT21 primer (Metabion, Planegg, Germany). Transcription of *PR-1* and *ICS1* genes was determined using real-time qPCR. Gene transcription values were normalized to *TIP41*. The primers used are listed in the Supplementary Table S1.

### 4.5. Photosynthetic Parameter Analysis

Plants were put in the dark for 15 min, and then the photosynthetic parameters were measured using FluorCam Handy FC 1000-H (PSI, Drasov, Czech Republic). Images of whole plants were taken. Chlorophyll fluorescence images were analysed using FluorCam 7.0 (PSI) software. Non-photochemical quenching (NPQ) was calculated as (Fm − Fm’)/Fm’ and maximum quantum efficiency of PS II photochemistry (QY) was calculated as Fv/Fm. Fm and Fm´ are the maximal fluorescence level from the dark-adapted and light-adapted leaf, respectively, and Fv is variable fluorescence from the dark-adapted leaf [52].

### 4.6. Statistical Analysis

All experiments were done in three biological repetitions. For soil-grown plants, *n* = 24; for in vitro grown plants, *n* ≥ 10 for each genotype. Graphs display analysis of all values together, unless stated otherwise. Student’s *t*-test and one-way ANOVA with Tukey’s post hoc test were applied for the comparisons, *p* < 0.05. Correlation analysis was done using R software, *Corrplot* package [53]. Pearson coefficients were quantified, and only the values that passed the *p* < 0.05 threshold are displayed.

## 5. Conclusions

In this study, we introduced a new tool for studying the role of SA role in plants, the Arabidopsis “SA collection”. It provides a robust tool benefitting from the distinct origin of the modulated SA pathway in *Arabidopsis thaliana*. The effective usage of the SA collection was demonstrated by phenotyping under different growing conditions, in soil and in vitro, using several light regimes. First, our data confirmed the correlation of SA content and expression of SA-related genes in different cultivation setups. Second, we clearly showed that SA is responsible for the regulation of rosettes, but not growth. Additionally, the SA collection revealed that a high basal SA content makes rosettes more sensitive to light. Surprisingly, we reassessed that *pmr4* mutant is not SA-OA under basal growing conditions. The presented SA collection is a starting point for future research trying to determine the roles of SA in response to environmental changes and to shed light on the complexity of SA-triggered signalling.

## Figures and Tables

**Table 1:** Selected Arabidopsis mutants with potentially affected salicylic acid (SA) signatures.

Mutant Name	Targeted Gene		Targeted Process	Reference
*cpr5*	*CPR5*	Constitutive Expression of Pathogenesis-related genes 5	Constitutive expression of pathogenesis-related genes 5	Yoshida et el. 2002 [20]
*bon1-1*	*BON1*	BONZAI 1	Negative regulator of cell death, defence responses and several R genes	Li et al. 2007 [15]
*acd6*	*ACD6*	Accelerated Cell Death 6	Dose-dependent activation of defence signalling, accelerated cell death observed	Rate et al. 1999 [21]
*pi4kβ1β2*	*PI4Kβ1, PI4Kβ2*	Phosphatidylinositol-4-kinase β1 and β2	Second messenger, phosphatidyl inositol-4-phosphate production	Preuss et al. 2006 [22]
*fah1fah2*	*FAH1, FAH2*	Fatty acid5-hydroxylase 1 and 2	Fatty acid hydroxylation	Konig et al. 2012 [19]
*edr2-6*	*EDR2*	Enhanced Disease Resistance 2	Negative regulation of cell death	Vorwerk et al. 2008 [23]
*exo70B1-1*	*EXO70B1*	Exocyst Complex Component EXO70B1	Endomembrane trafficking	Kulich et al. 2013 [18]
*pmr4-1*	*CALS12*	Callose Synthase 12	Pathogen-induced callose synthesis	Nishimura et al. 2003 [24]
*sid2* *pi4kβ1β2*	*ICS1, PI4Kβ1, PI4Kβ2*	Isochorismate synthase 1, phosphatidylinositol-4-kinase β1 and β2	SA biosynthesis, second messenger inositol-1,4,5-trisphosphate production	Sasek et al. 2014 [25]
*NahG* *pi4kβ1β2*	*NahG, PI4Kβ1, PI4Kβ2*	SA hydroxylase, phosphatidylinositol-4-kinase β1 and β2	SA degradation, second messenger inositol-1,4,5-trisphosphate production	Sasek et al. 2014 [25]
*NahG* *edr2-6*	*NahG, EDR2*	SA hydroxylase, enhanced disease resistance 2	SA degradation, negative regulation of cell death	Vorwerk et al. 2008 [23]
*bon1-1* *snc1-11*	*BON1, SNC1*	BONZAI 1, Suppresssor npr1-1, constitutive 1	*bon1-1* crossed to the *snc1-11*, loss-of-function point mutation of the *SNC1*	Li et al. 2007 [15]
*sid2*	*ICS1*	Isochorismate synthase 1	SA biosynthesis	Wildermuth et al. 2001 [8]
*NahG*	*NahG*	SA hydroxylase	SA degradation	Nawrath and Metraux 1999 [26]

## Supplementary Materials

Supplementary materials can be found at https://www.mdpi.com/1422-0067/20/24/6365/s1.

## Abbreviations

SA	Salicylic acid
SA-OA	Salicylic acid overaccumulating mutants
ICS1	Isochorismate synthase 1
PR1	Pathogenesis related protein 1
SD	Short day conditions
LD	Long day conditions
