# Supplementary material for: “Salicylic Acid Mutant Collection” as a Tool to Explore the Role of Salicylic Acid in Regulation of Plant Growth under a Changing Environment
Source: Int J Mol Sci. 2019 Dec 17;20(24):6365. doi: 10.3390/ijms20246365 (PMC6941003; doi:10.3390/ijms20246365)
Supplement: Supplementary file 1 [file ijms-20-06365-s001.pdf]

International Journal of *Molecular Sciences*

MDPI

Supplementary Material

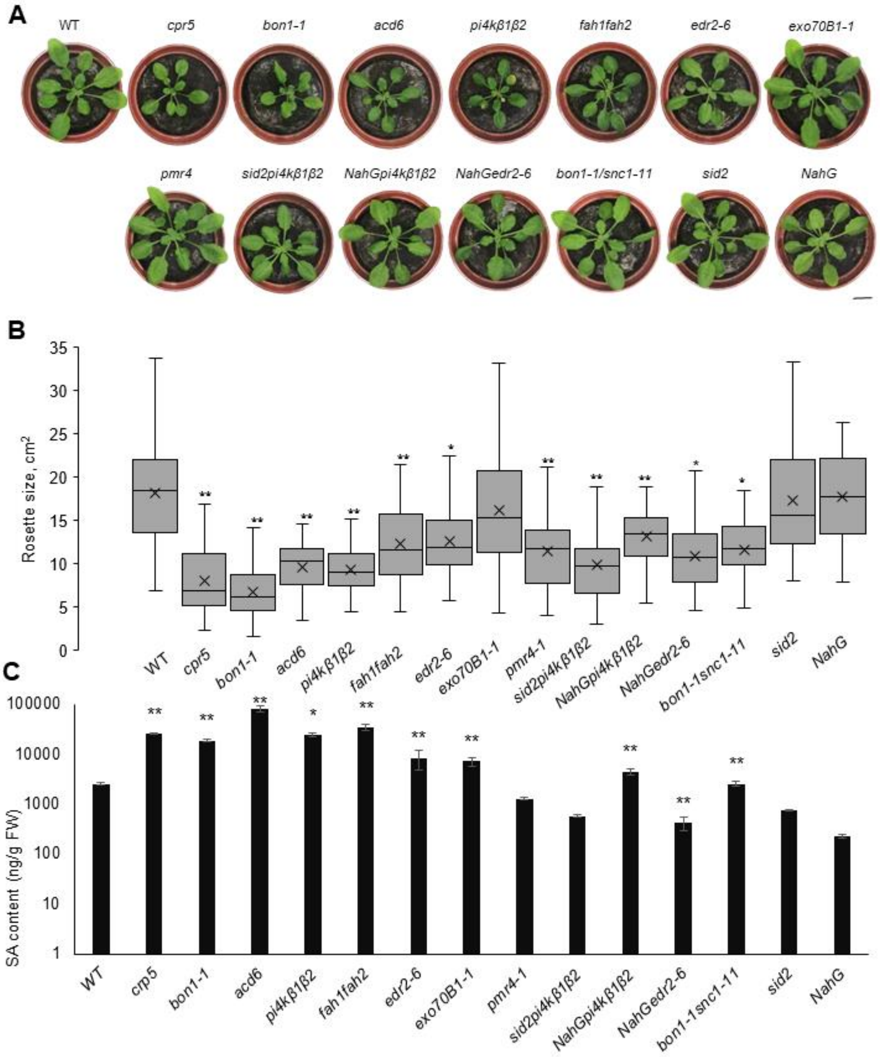

Supplementary Figure S1. Rosette size and salicylic acid (SA) content of plants cultivated under short-day conditions. (**A**) Representative images of 4 week old plants cultivated at SD conditions: 22 °C, 10 h light/14 h dark. (**B**) Rosette size. "Immune-related" mutants presented in blue boxes; "phospholipids-related" in red; callose synthase *pmr4* in pink; "reverted" double/triple mutants in yellow and SA-deficient in green. Data are from three biological replicates, $n \geq 70$.Central line of the boxplot represents the median occupancy, cross represents the mean, bottom and top edges of the box are 25 and 75% of distribution and the ends of whiskers are set at 1.5 times the interquartile range. Values outside this range are shown as outliers. Data are from three biological replicates, $n \geq 70$. (**C**) SA content in the leaves. $n = 4$. Asterisks indicate variants that are different from WT, one-way ANOVA with Tukey's HSD post hoc test, * $p < 0.05$, ** $p < 0.01$.

A

	LD	SD	GH
Growing conditions	Long day	Short day	Greenhouse
Humidity	70%	70%	-
Light intensity	110µE	110µE	-
Temperature	22⁰C	22⁰C	-
Light regime	16h light/ 8h dark	10h light/ 14h dark	16h light/ 8h dark

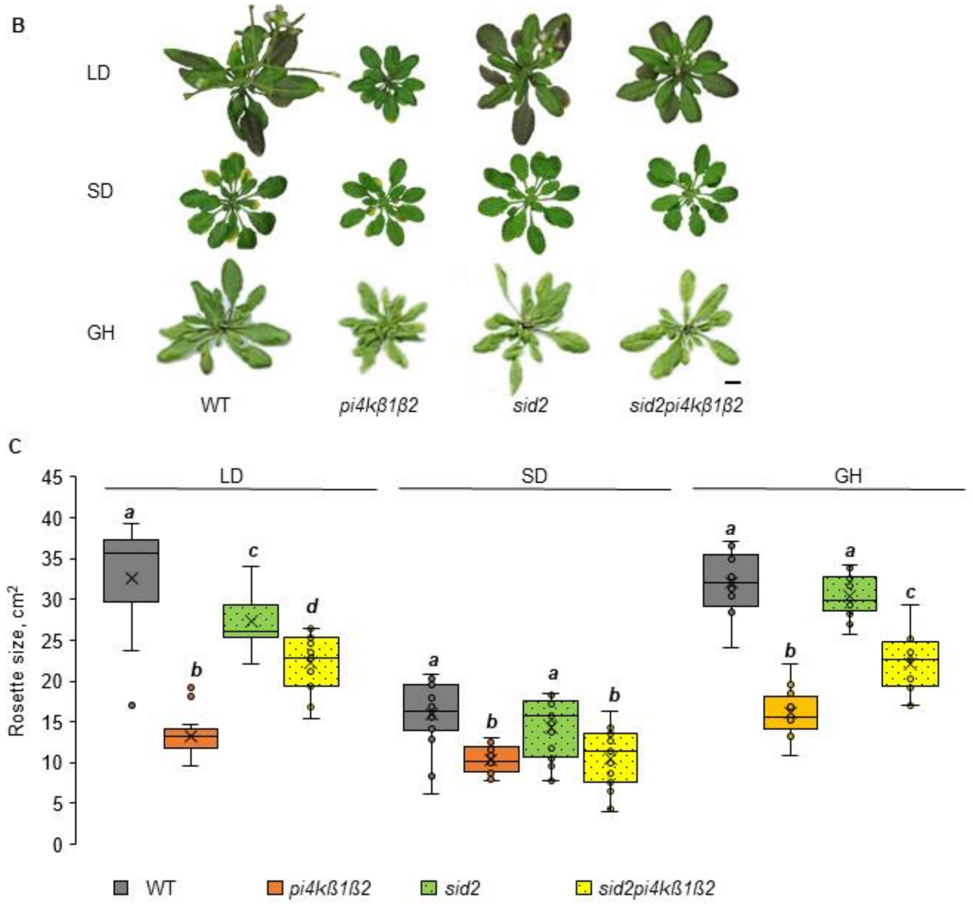

Supplementary Figure S2. Effect of cultivation conditions on rosette size of the *pi4kβ1β2* and *sid2pi4kβ1β2* mutants. (**A**) Conditions of plant cultivation: LD (long day), SD (short day) and GH (greenhouse). (**B**) Representative images of 5 week old plants cultivated in different setups, bar 1 cm. (**C**) Rosette size of the studied mutants grown under different conditions. Different letters indicate variants that were significantly different in every growing condition; one-way ANOVA with Tukey's HSD post hoc test, $p \leq 0.05$. Data are from three biological replicates, $n \geq 12$. Central line of the boxplot represents the median occupancy, cross represents the mean, bottom and top edges of the box are 25% and 75% of distribution and the ends of whiskers are set at 1.5 times the interquartile range.

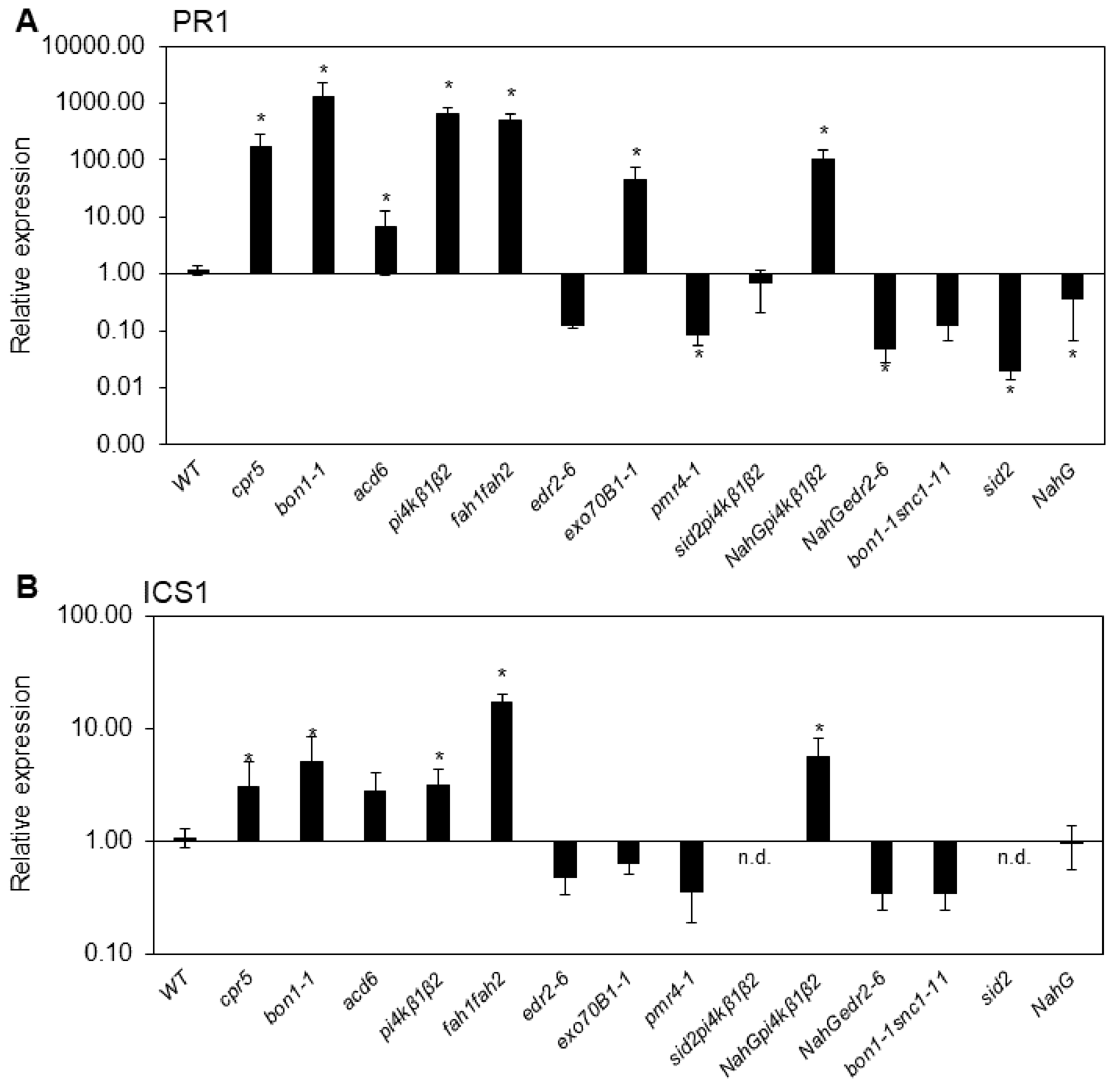

Supplementary Figure S3. Transcription of *ICS1* and *PR1* in soil-grown plants cultivated under a short-day light regime. Samples were collected from four 4 week old plants. Values were normalized to WT in the respective conditions. *TIP41* was used as a reference gene. Asterisks indicate values different from WT, *t*-test, $p < 0.05$, $n = 4$.

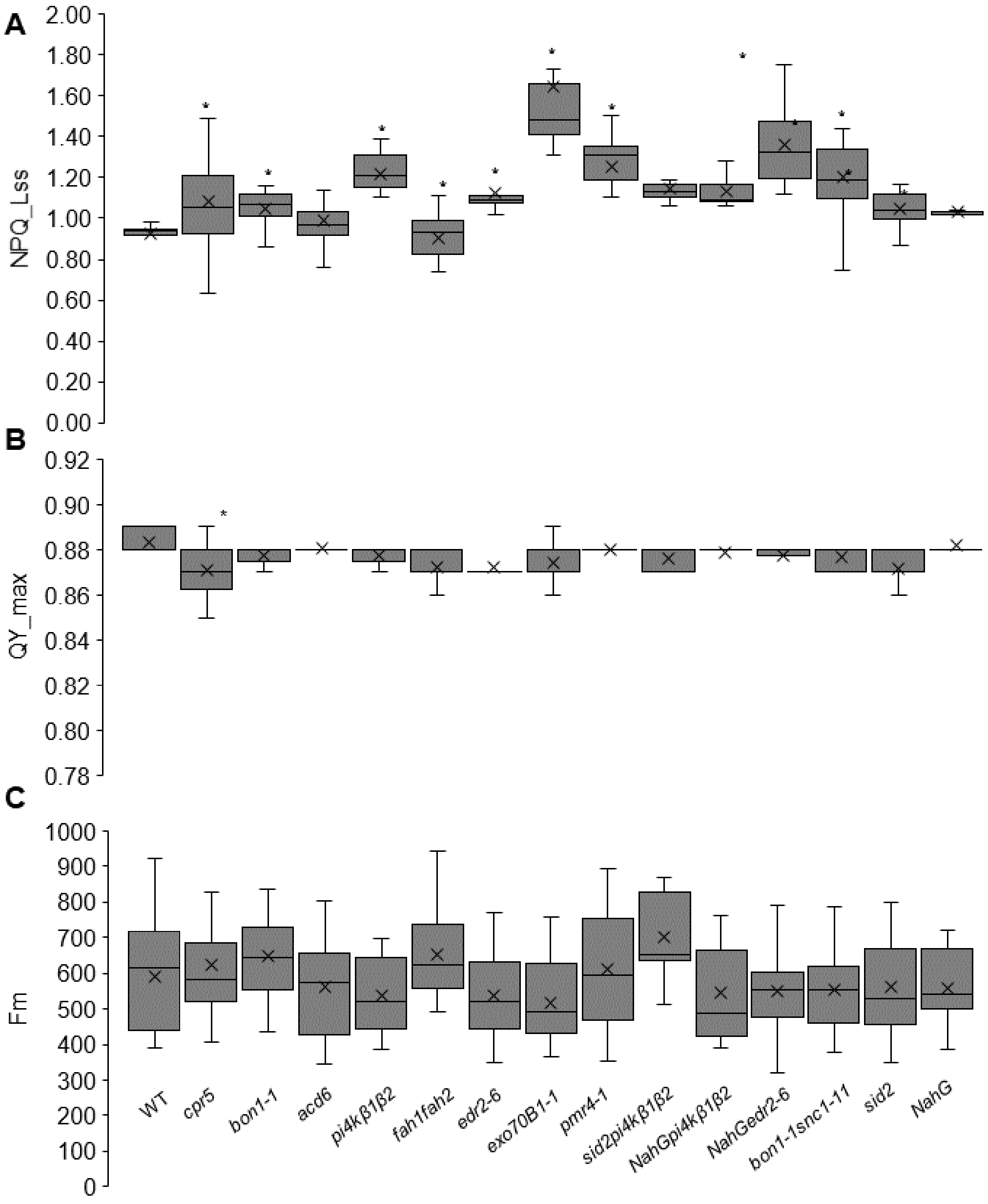

Supplementary Figure S4. Photosynthetic parameters of the studied mutants. Four week old plants were cultivated at 22 °C, 10 h light/ 14 h dark. (**A**) NPQ-Lss. (**B**) QY_max. (**C**) Fm. Central line of the boxplot represents the median occupancy, cross represents the mean, bottom and top edges of the box are 25% and 75% of distribution and the ends of whiskers are set at 1.5 times the interquartile range. Values outside this range are shown as outliers. * variants that are different from WT, one-way ANOVA with Tukey's HSD post hoc test. Data are from three biological replicates, $n \geq 70$.

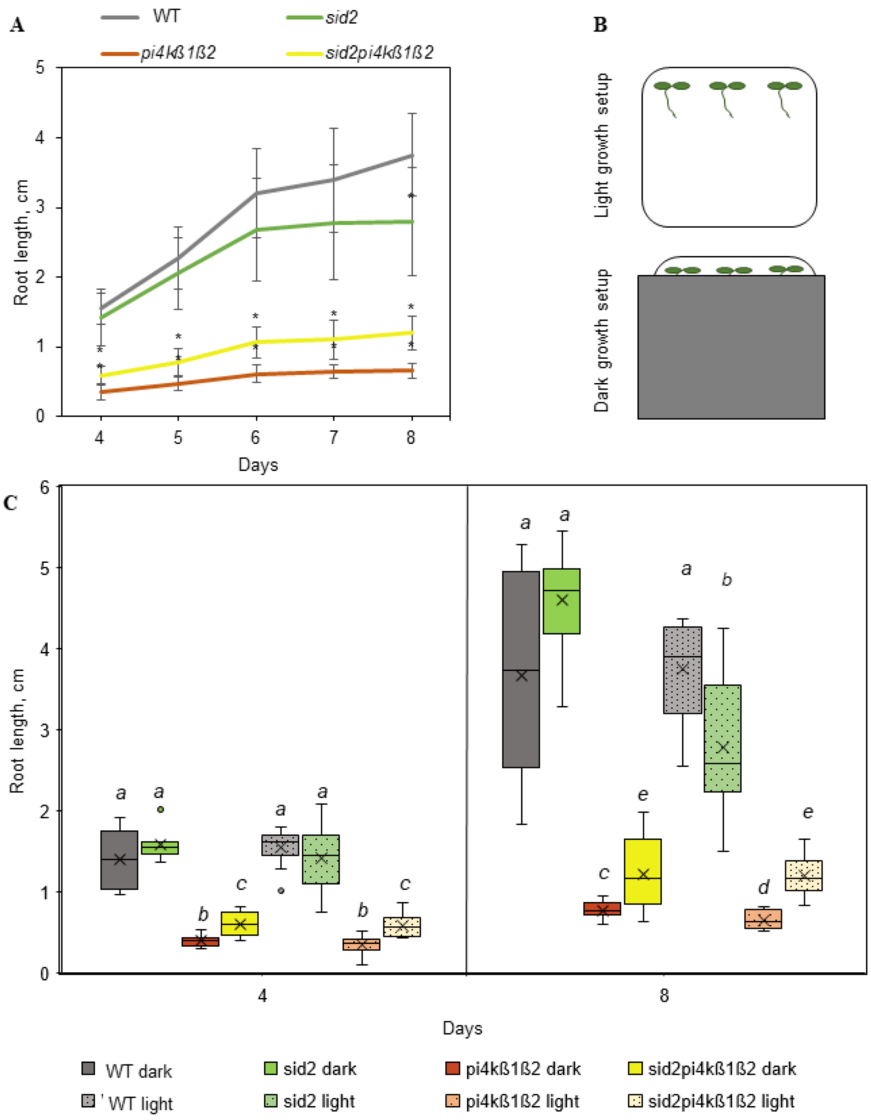

Supplementary Figure S5. Effect of light on primary root elongation of the *pi4kβ1β2* and *sid2pi4kβ1β2* mutants grown in vitro under long day conditions. (**A**) Dynamics of primary root growth under long-day conditions. * variants that are different from WT, one-way ANOVA with Tukey's HSD post hoc test, $n \geq 10$. (**B**) Light and dark root setup. (**C**) Length of primary root grown in light or dark root setup at 4 and 8 days. Central line of the boxplot represents the median occupancy, cross represents the mean, bottom and top edges of the box are 25% and 75% of distribution and the ends of whiskers are set at 1.5 times the interquartile range. Differences calculated separately in each group, 4 and 8 days respectively. Letters correspond to significant differences among groups, $n \geq 10$.

Supplemental Table S1. Primers.

Gene	Accession	Forward Primer	Reverse Primer
TIP4 1	AT4G34270	GTGAAAACTGTTGGAGAGAAGCAA	TCAACTGGATACCCTTTCGCA
PR-1	AT2G14610	AGTTGTTTGGAGAAAGTCAG	GTTCACATAATTCCCACGA
ICS1	AT1G74710	GCAAGAATCATGTTCCTACC	AATTATCCTGCTGTTACGAG

Supplemental Table S2. SA content (ng/g FW) of the soil-grown plants.

	Short day			Long day		
**WT**	**251,380**	**±**	**15,930**	514,591	±	180,327
NahG	225,75	±	17,78	260,36	±	90,84
sid2	741,55	±	24,01	215,81	±	50,57
pmr4	1275,86	±	50,99	994,22	±	107,14
pi4kβ1β2	23851,29	±	1950,85	9410,93	±	982,55
NahGpi4kβ1β2	4458,48	±	659,26	455,29	±	130,99
sid2pi4kβ1β2	567,54	±	31,92	172,26	±	28,88
crp5	25511,32	±	800,46	29118,68	±	17116,81
bon1-1	18437,73	±	964,35	32831,49	±	19690,02
bon1-1snc1-11	2504,99	±	264,97	612,13	±	141,04
edr2-6	8375,98	±	3633,38	2439,16	±	605,55
edr2-6nahG	422,06	±	124,73	1872,03	±	1091,57
fah1fah2	33314,76	±	4732,62	64289,30	±	42158,94
exo70B1-1	7137,93	±	1340,52	1312,63	±	292,41
acd6	80154,83	±	10881,94	61875,90	±	7018,37
